# Supplementary material for: Applied aerial spectroscopy: A case study on remote sensing of an ancient and semi-natural woodland
Source: PLoS One. 2021 Nov 15;16(11):e0260056. doi: 10.1371/journal.pone.0260056 (PMC8592455; doi:10.1371/journal.pone.0260056)

**S2 Fig. Stitched orthomosaic images of dead trees**

(a) 17 September 2020


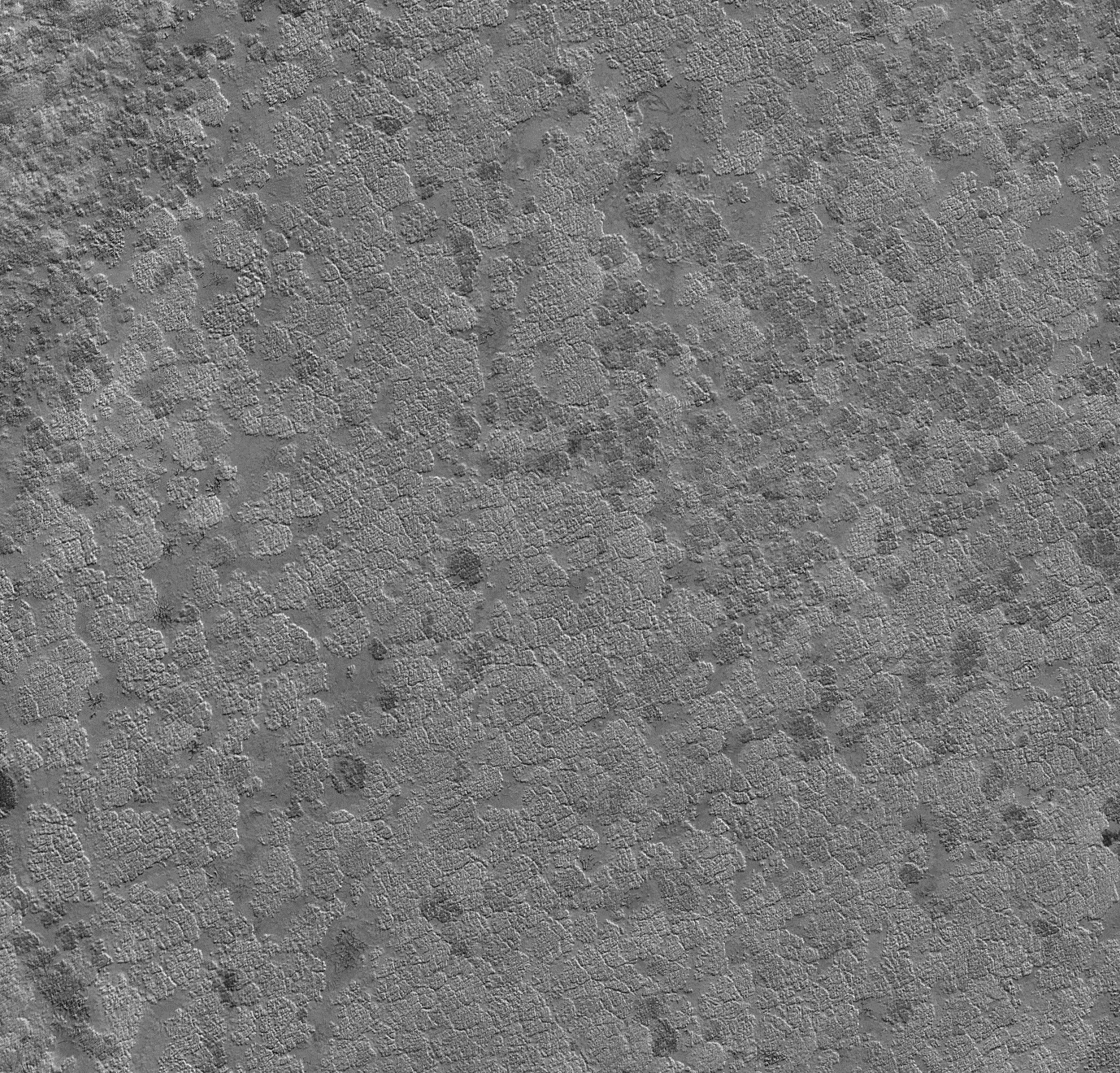


(b) 18 September 2020


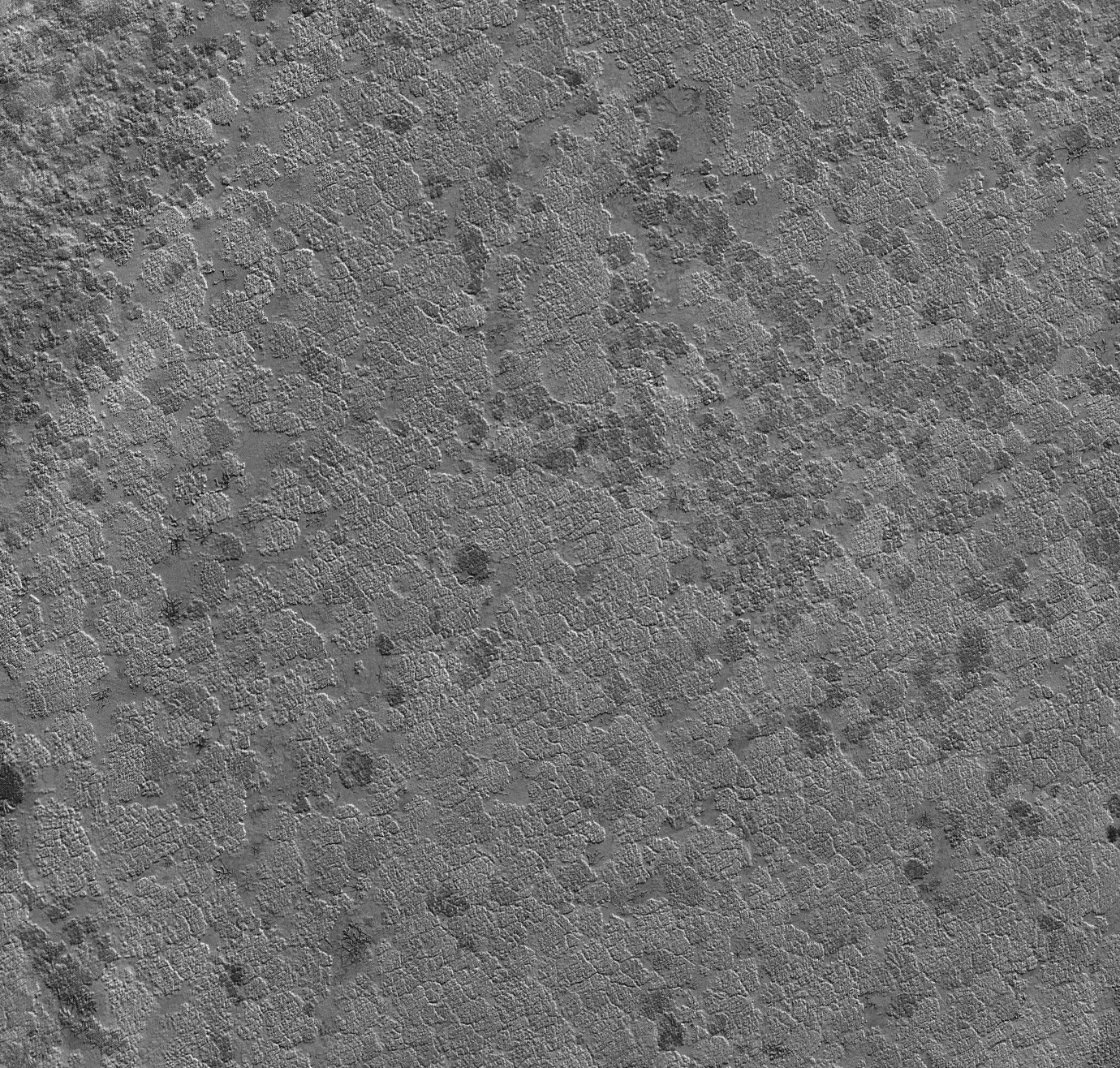


(c) 28 September 2020


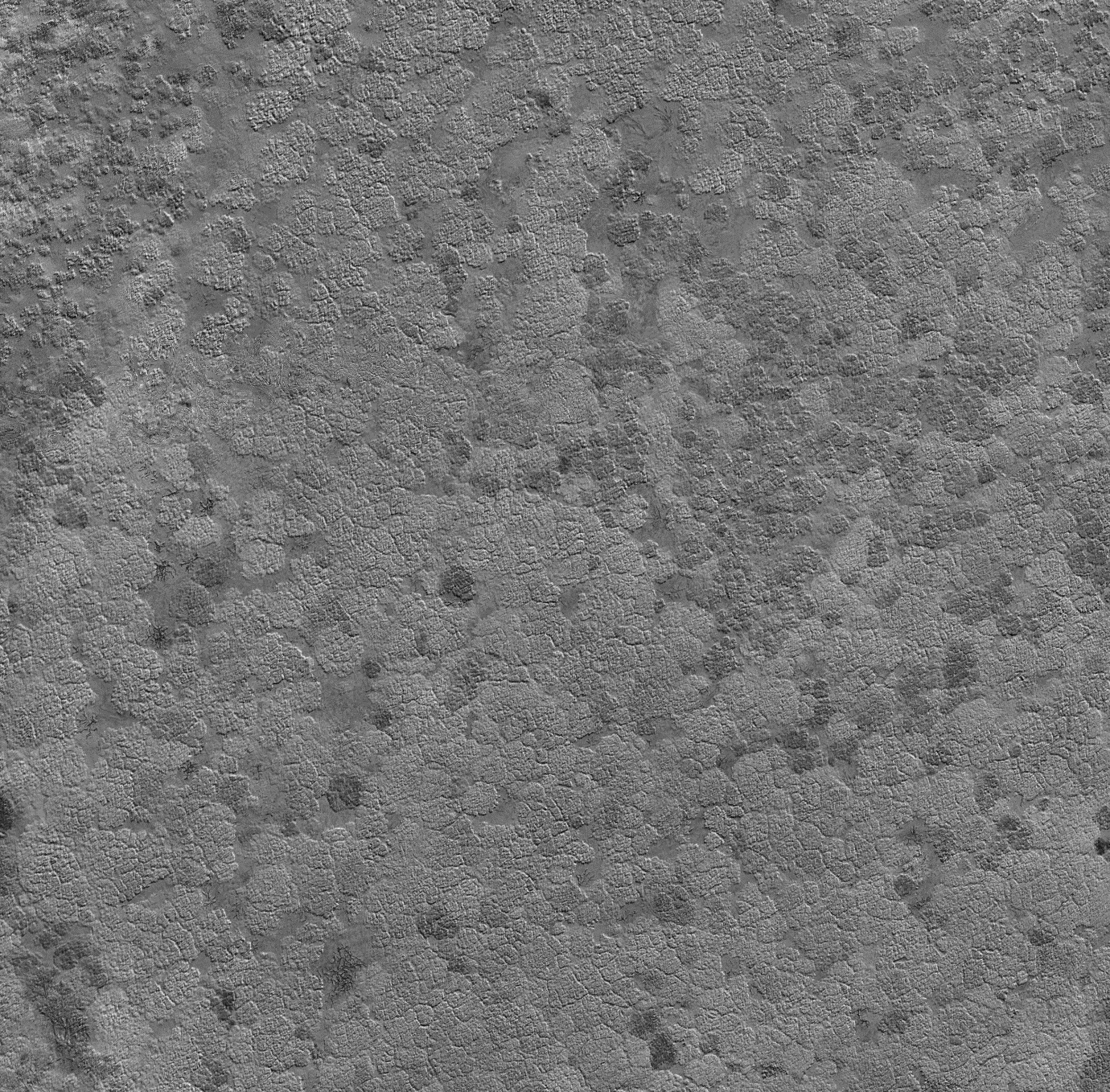


(d) 8 October 2020


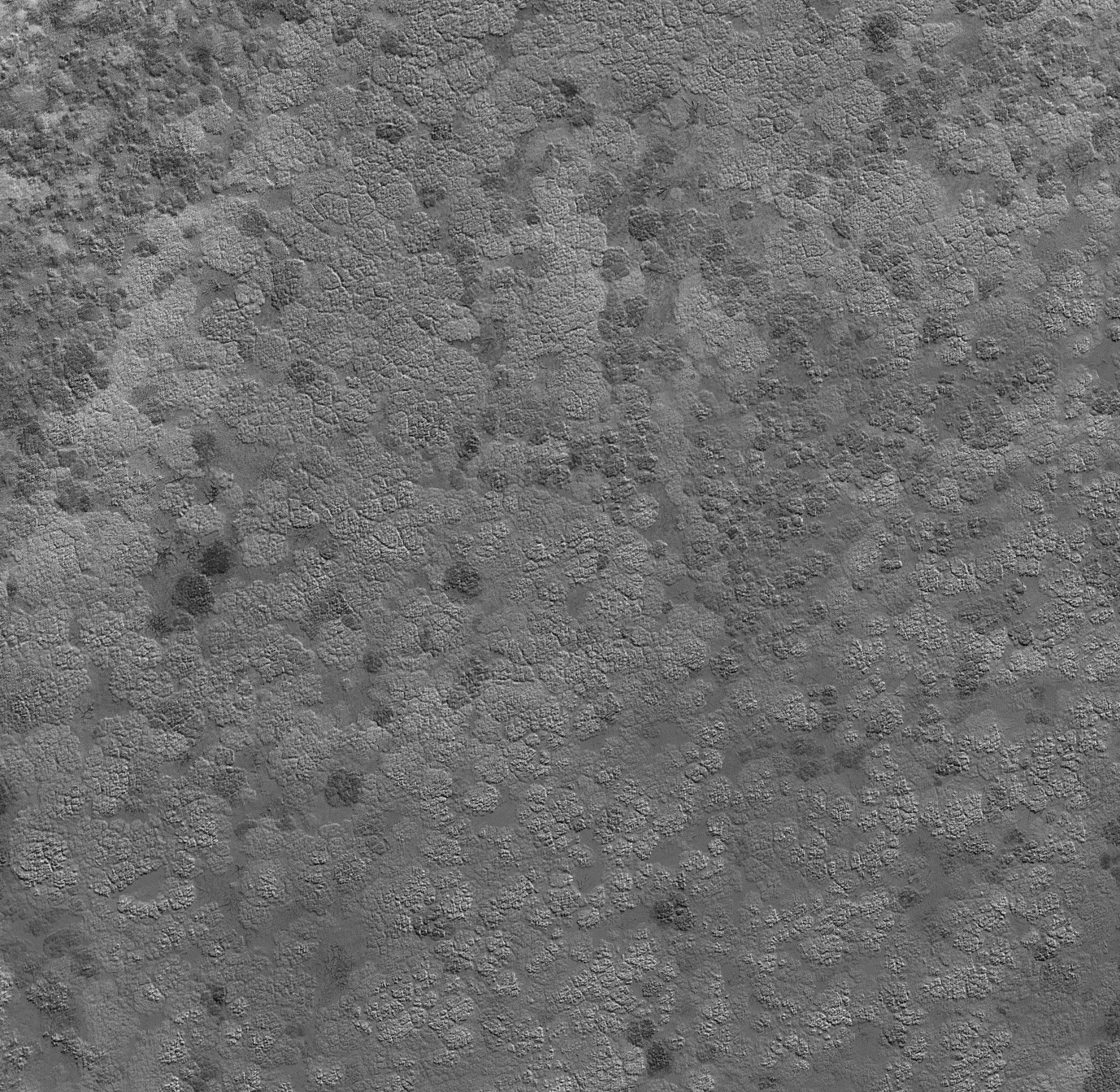


(e) 14 October 2020


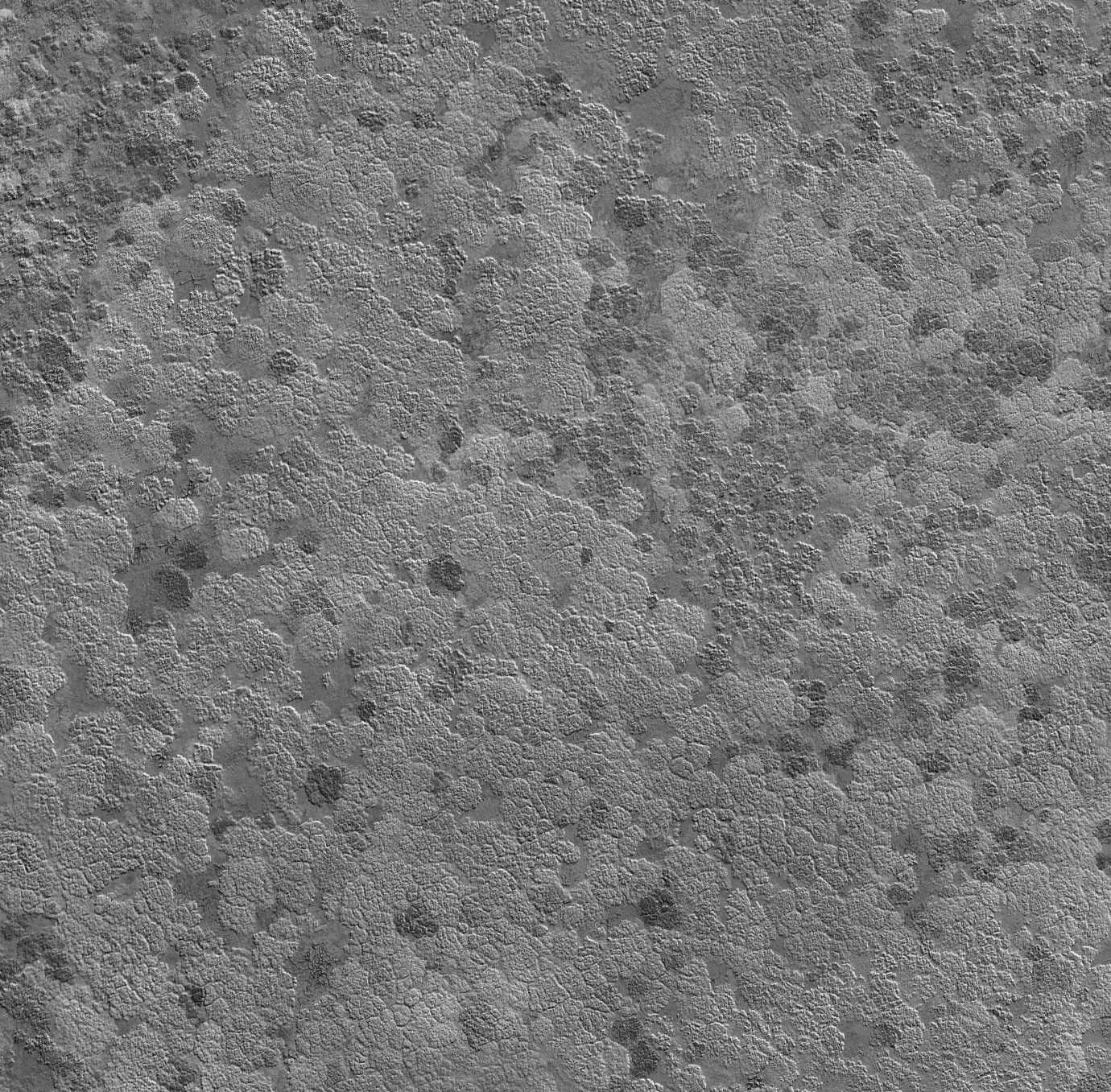

Supplement: S2 Fig — (DOCX) [file pone.0260056.s002.docx]
