# Supplementary material for: Applied aerial spectroscopy: A case study on remote sensing of an ancient and semi-natural woodland
Source: PLoS One. 2021 Nov 15;16(11):e0260056. doi: 10.1371/journal.pone.0260056 (PMC8592455; doi:10.1371/journal.pone.0260056)
Supplement: Attachment — Submitted filename: response to reviewers.odt [file pone.0260056.s004.odt]

Responses to reviewer’s comments

Comments to the Author

1. Is the manuscript technically sound, and do the data support the conclusions?

The manuscript must describe a technically sound piece of scientific research with data that supports the conclusions. Experiments must have been conducted rigorously, with appropriate controls, replication, and sample sizes. The conclusions must be drawn appropriately based on the data presented.
Reviewer #1: No
Reviewer #2: Yes
Reviewer #3: Partly
Reviewer #4: Partly

Author response: We have thoroughly revised the manuscript to identify the novelty of the research presented. This is evidenced in the track changes manuscript (submitted) along with the new (revised) figures: 6 in total.

2. Has the statistical analysis been performed appropriately and rigorously?
Reviewer #1: No
Reviewer #2: Yes
Reviewer #3: N/A
Reviewer #4: No

Author response: were appropriate statistically information is included; see for example Table 2 and its footnote.
3. Have the authors made all data underlying the findings in their manuscript fully available?

The PLOS Data policy requires authors to make all data underlying the findings described in their manuscript fully available without restriction, with rare exception (please refer to the Data Availability Statement in the manuscript PDF file). The data should be provided as part of the manuscript or its supporting information, or deposited to a public repository. For example, in addition to summary statistics, the data points behind means, medians and variance measures should be available. If there are restrictions on publicly sharing data—e.g. participant privacy or use of data from a third party—those must be specified.
Reviewer #1: Yes
Reviewer #2: Yes
Reviewer #3: No
Reviewer #4: No
Author response: all data is included in the submission.

4. Is the manuscript presented in an intelligible fashion and written in standard English?

PLOS ONE does not copyedit accepted manuscripts, so the language in submitted articles must be clear, correct, and unambiguous. Any typographical or grammatical errors should be corrected at revision, so please note any specific errors here.
Reviewer #1: No
Reviewer #2: Yes
Reviewer #3: Yes
Reviewer #4: Yes
Author response: the grammatical representation of the text has been updated and revised.

5. Review Comments to the Author

Please use the space provided to explain your answers to the questions above. You may also include additional comments for the author, including concerns about dual publication, research ethics, or publication ethics. (Please upload your review as an attachment if it exceeds 20,000 characters)
Reviewer #1: General comments:
The work aims at identifying/classifying tree species using drone (UAV) multispectral imagery across a 17 ha woodland. While this is an important and challenging topic to research on, I feel that the work does not bring any novelty in this topic.
Author response: The novelty aspect has been brought out more in the revised text. The key aspects are the new NDSI with thresholding to identify tree species. This is a much simpler approach that has been used by others, using a low cost UAV.

The authors comment in L. 459-460 that ‘there are currently many studies reporting the use of UAVs to identify and quantify tree species’. This is true and it brings a challenge in developing a new method/technique among so many published studies in the field. In fact, the authors did not identify the state of the art in tree species classification (or identification) with UAV imagery (or any other very high spatial resolution remote sensing imagery). Therefore, it is not clear the research gap that needs to be addressed. It is difficult to grasp the novelty of the work.
Author response: This whole aspect has been revisited, and includes detailed reasoning and justification why the use of a low cost UAV with MSI does provide a novel way to investigate a ASNW.

The Methodology lacks a detailed description and explanation (why) of method choices, which would allow others to replicate the work. Also, a classification would entail a robust quantitative assessment of its accuracy – which is missing.
Author response: The quantitative assessment is re-emphasised in the main text as well as the footnote in Table 2. The revised manuscript addresses many of these queries.

In the title, the authors use the word ‘spectroscopy’ which is generally associated with hyperspectral data – this is clearly not the case.

Author response: unfortunately, we disagree, as a spectroscopist who is used to it use for many applications from flame photometry (where a single filter is used to identify sodium) through to ICP-AES techniques. In this case using RGB, red edge and NIR is spectroscopy.


Specific comments:
L.16-18: “This derived data was corroborated by a 17 statistically valid ground-level field study, over an area of 10,800 m2, that identified oak 18 (47%), silver birch (46%) and dead trees (7.4%).”
This sentence is confusing. I suppose the authors want to say something about the accuracy but this is not clear.
Author response: sentence modified taking into account this comment

L. 19-29: ‘In addition, it was noted that a single UAV flight took 22 minutes as compared to the ground-level field study data that took approximately 4-5 hours.’
This sentence doesn’t seem relevant for an abstract. Nevertheless, despite the ground surveying taking longer that the UAV one, the field identification is ready as soon it is done. The UAV data processing may take several days.
Author response: This has been removed from the abstract and revised elsewhere as well. We have also emphasised the low computing power required for our approach. UAV data processing does not take days.

Abstract: It is not clear the novelty, methods, results and conclusions in the abstract. Needs re-thinking. Also, there is no mention of aerial spectroscopy in the abstract. In fact, the title says spectroscopy but the abstract uses multispectral. When I read aerial spectroscopy, I thought about hyperspectral data.
Author response: This has been re-written and the key novelty aspects added.

Introduction: The state of the art in tree species classification with UAV imagery (or any other very high spatial resolution remote sensing imagery) is not brought up in the Introduction. Therefore, it is not clear the research gap that needs to be addressed.
The remote sensing-related topics are discussed in a superficial way.
Also, there are too much information about woodlands (and its history) in the Introduction. This should be presented more concisely in the Introduction. If the authors want to bring all such information to the readers, they could, perhaps, create a new sub-section in the Methodology.
Author response: this section has been totally revised, as can be seen on the track changes copy.

L. 173: ‘2. Experimental’ This is not usual. I would expect ‘Methodology’.
Author response: agreed and done

Figure 2: The work would benefit of a better study area map, showing not only the woodland of interest, but also its location within the UK.
Author response: agreed and done.

Figure 3: A bar-like scale would give a better idea of size for readers.
Author response: agreed and done.

L. 206: ‘2.3 Field Data Analysis’. What was the sampling strategy used? And why?
Author response: As indicated in the text a whole area was investigated and mapped by individuals. The area selected was confirmed to be statistically important (see table 2 footnote).

L. 213: ‘total field site of 10,800 m2 was covered’. Why this area? It is different from the area covered by the UAV. Why?
Author response: As indicated in the text a whole area was investigated and mapped by individuals. The area selected was confirmed to be statistically important (see table 2 footnote).


L. 221-228: ‘2.4.1 Photogrammetric Processing’ What were the Agisoft parameters chosen and why? How was the georeferencing done and which positional accuracy was achieved?

Author response: more details have been added.

L. 330-331: ‘Images derived from the new NDSI = (PC1-PC3)/(PC1+PC3), were stitched using Agisoft Metshape Professional software to build an orthomosaic image.’ Why not producing an orthomosaic first and then carry out any further image processing?

Author response: Our procedure was done in that specific order as the resolution was not retained by the Agisoft software when done the other way around. We have added a new revised Figure (5) that outlines the whole process.

L. 459-460: ‘Although there are currently many studies reporting the use of UAVs to identify and quantify tree species’
Exactly, there are many indeed. So, the authors need to review as many as possible of such studies, identify what was already done, identify what needs to be done and, then, clearly identify the big novelty (research gap) of this study in the Introduction section and how it advances what is already published.
Author response: We have extensively revised the text to highlight the novelty of our approach.
Reviewer #2: This manuscript used remote sensing technologies to monitor ancient and seminatural woodland. The idea is interesting and the result is basically satisfactory. However, some other problems in the manuscript are still concerned in the following
Author response: We now hope we have addressed all concerns of all 4 reviewers by modifying both the text and figures.
Reviewer #3: I have my detailed comments written in a word file, please see the attached file.
I suggested major revision because it is not so clear the results of the research, and the figures are not well-drawn.

Author response: We now hope we have addressed all concerns of all 4 reviewers by modifying both the text and figures. The new figures address this reviewers concerns.

Reviewer #4: The paper needs to revised to explain the true sense of Applied aerial spectroscopy. The work lacks in statistical inference beyond the pictorial representation of the results. The work is required to be added to tabular results. One section is required to be added for societal applications. Apart from these new references is required to be added.
Author response: We now hope we have addressed all concerns of all 4 reviewers by modifying both the text and figures. Statistical data has been added in both the text and table 2 footnote.


6. PLOS authors have the option to publish the peer review history of their article (what does this mean?). If published, this will include your full peer review and any attached files.


If you choose “no”, your identity will remain anonymous but your review may still be made public.


Do you want your identity to be public for this peer review? For information about this choice, including consent withdrawal, please see our Privacy Policy.
Reviewer #1: No
Reviewer #2: No
Reviewer #3: Yes: Liping Li
Reviewer #4: Yes: Dr. Deepak Kumar

Editor summary
Specifically:
- The authors did not identify the state of the art in tree species classification (or identification) with UAV imagery (or any other very high spatial resolution remote sensing imagery).
- It is not clear the research gap that needs to be studied, representing the novelty of the work.
- The Methodology lacks a detailed description and explanation (why) of method choices.
- UAV data and field survey data were not obtained at a similar period of the year, which may influence the results.
- The work lacks in statistical inference beyond the pictorial representation of the results.

Author response: We now hope we have addressed all concerns of all 4 reviewers by modifying both the text and figures.

Reviewer:
Dear Claudionor,
With a small dataset (73,934 m2 of UAV, and 10,800 m2 ground study), this research studied the classification of dead trees, oak and silver birch trees with the NDVI indicator and the PCA extracted indicators. It is a good exploration of the species classification method with drone data. But there are still some points not clear. The authors could modify the paper in the later stage.
1. There are several band information from the multispectral data. You could try these before you used PCA method. Please state why not and please also state the rationality of the NDVI method used for the PCA result. And please explain more clearly why you use PC1 & PC2, PC1 & PC3 for the analysis.
Author response: We now hope we have addressed this concern by adding more detailed explanation to the text, including the justification for the choice of the PC’s.

2. The UAV data and the field survey data were not obtained in a similar time of a year, please explain the influence of this on the results. And the fieldwork, although in very small area, was conducted in a very short time by only two people, I was wondering how it was done. 
Author response: We clearly indicate when the data was obtained. Winter for the UAV work would have been unsuitable as the leaves are no longer present. Also, as the trees do not change physically we were able to revisit later in the year. [I walk in the same woods every day as part of an exercise programme so no deforestation had taken place.]. Details are in the Table 2 footnote explaining the procedure, as well as the Methodology.

3. Some figures are not clear. For example, Fig. 9, the outline was not marked for dead trees. And the outlines of oak and silver birch trees were not precise, and we could even say it seemed very coarse. And also Fig. 10, 
Author response: totally agree, the figures have been re-done.

These outlines, for example, the places with red ones I drew, are so not precise. And why do not you use just one line for the border between two plant species?
Author response: totally agree, the figures have been re-done.

4. With your method explored, why you still manually draw the outline, i.e., the classification, why do not you use the auto result of the program with your model? Currently we could just see by the eyes part of the results, and I think the auto interpretation from your methods is the very important point you should stress in your paper, which is also the significance of your research.  
 Author response: totally agree, the figures have been re-done, and the text modified.

 And some specific points:
Line 16: in the abstract and also the main body of the text, please leave the same digits of the numbers, for example, 1.0% or 1%. 
Author response: agreed and done.
Line 41: gradually less expensive.
Author response: agreed and done.
Line 73: “protect the destruction”? Not right expression?
Author response: agreed and modified.

Line 78-82: please check the area you described of the woodland, better with either ha, or m2, but not a lot of mixes of the two.
Author response: agreed. We have retained hectares (but included m2 in brackets).

Line 116: “the interest in the use of …” was raised? And delete the “Therefore” sentence?
Author response: agreed and done.

Line 163: the aim of (c) is not proper, is the aim of the ground data investigations being confirming your drone data precision?
Author response: agreed and done.

Line 164: precision à precise?
Author response: agreed and done.

Line 167: “Any plan” --- > this plan
Author response: agreed and done.

Line 173: “Experimental” --- > Materials and methods
Author response: agreed and done.

Line 178: “hectares” or ha, better use the same in the whole file.
Author response: agreed and done.

Line 189: I am not sure whether this “ (DJI Phantom 4, Colena Ltd., North Shields, UK)” is the right citation. Is “Colena Ltd.” the company that supplies the drone? As far as I know this drone was produced in China.
Author response: agreed and done. Colena are the supplier.

Line 206: “Field Data Analysis” --- > Field data analysis , and line 221, 222 or you change the other titles.
Author response: agreed and done.

Line 234: in --- > was 
Author response: agreed and done.

Line 277: Is “% variance” the right expression?
Author response: agreed it should be % cumulative.

Line 345: It is not clear how the precision assessment was realized.
 Author response: by comparing UAV data and the ground study.

And, please check the legend of Fig. 8. 
Author response: agreed and modified.

There is big blank space in some figures, for example, Fig. 11.
Author response: not sure, but has been redone.

 
 
 Reviewer
This manuscript used remote sensing technologies to monitor ancient and seminatural woodland. The idea is interesting and the result is basically satisfactory. However, some other problems in the manuscript are still concerned in the following: 
1. The keywords are missing. 
Author response: unclear as they were present all the time. Original manuscript lines 26-27.

2. The organization of this manuscript should be added to the end of the introduction. 
Author response: not sure what this means?

3. In the experiments, could the authors show more visual results of classification of trees species? 
Author response: agreed and done.

4. How is the time cost of the proposed method? 
Author response: not sure we can quantify as it would involve salary information. 

5. In this sentence, “In addition, the quality of satellite images is susceptible to weather, such as, cloud coverage and atmospheric absorption which needs to be corrected”, the references should be included, such as “DOI: 10.1109/IGARSS.2009.5417837”, “DOI: 10.1109/TGRS.2014.2307354”…
Author response: agreed and more / different references have been added.
