# Supplementary material for: Applied aerial spectroscopy: A case study on remote sensing of an ancient and semi-natural woodland
Source: PLoS One. 2021 Nov 15;16(11):e0260056. doi: 10.1371/journal.pone.0260056 (PMC8592455; doi:10.1371/journal.pone.0260056)
Supplement: Attachment — Submitted filename: response to reviewers and editor.docx [file pone.0260056.s005.docx]

PONE-D-21-23810

Editor responses:

Dear Claudionor,

**We acknowledge and provide the following information.**

**Methods section:** information with regard to the permits for the research have now been included. Previously it was in the acknowledgements.

**Competing interests**: we have done on the online submission.

**Figures 1, 2a and 2b. copyright issues:** Figure 1 has been deleted. Figure 2 a and b have been re-done using you direction to the USGS National Map viewer site. The figures in the text have been re-numbered accordingly.

**Reviewer 1:**

**Introduction**

Comment on L31-47: Noteworthy recent research of the applicability of multispectral UAV data for species recognition has already been mentioned with references from L128-143.

Comment on L93-115: The main difference between UAV, satellite and manned aircraft data has already been discussed, for example, manned aircraft and satellite data having low temporal resolution therefore cannot be collected multiple times within day (in L101-104). Whereas commentary on the UAV data being collected on regular intervals (in L104-106).

Comment on L146-167: Currently the most used approach, for example, NDVI is used for limited purposes. However, the purpose of the current study was to perform PCA and use the PCA results to derive a new NDSI which helped on the classification of native tree species which has been mentioned (in L146-167).

The main research gap is building up a suitable NDSI by incorporating multiple spectral bands for the specific native tree species to be classified in combination with thresholding method as the currently available method for tree species classification are time consuming requires training data sets as extensive machine learning and deep learning classification models are used. Whereas the method used in the current study is simple, feasible and less time consuming. Also, the newly build NDSI has been compared with NDVI in the results too

**Results and Discussion**

Comment on L418-419; The way how thresholding was performed is mentioned in the methodology (in L368-L380).

**Reviewer 2:**

Thank you.

**Reviewer 3:**

The sentence has been modified including removal of ‘are required’ (in L150-151).

**PLOS Data policy:** a link to the raw data has been added.
